# Understanding Hyperporphyrin Spectra: TDDFT Calculations on Diprotonated Tetrakis(*p*-aminophenyl)porphyrin

**DOI:** 10.1021/acs.jpca.1c06621

**Published:** 2021-10-29

**Authors:** Jeanet Conradie, Carl C. Wamser, Abhik Ghosh

**Affiliations:** † Department of Chemistry, UiT − The Arctic University of Norway, N-9037 Tromsø, Norway; ‡ Department of Chemistry, University of the Free State, P.O. Box 339, Bloemfontein 9300, Republic of South Africa; § Department of Chemistry, Portland State University, Portland, Oregon 97207-0751, United States

## Abstract

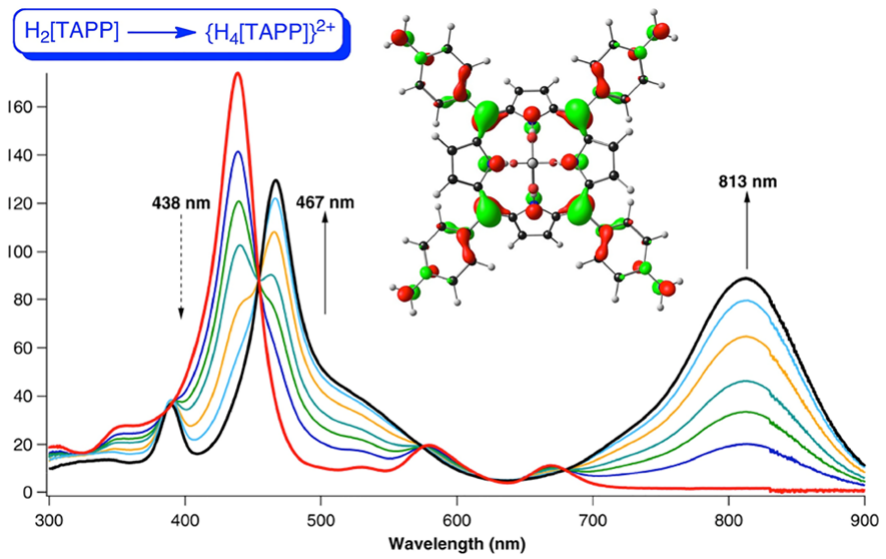

A detailed TDDFT study (with all-electron STO-TZ2P basis sets and the COSMO solvation model) has been carried out on the effect of diprotonation on the UV–vis–NIR spectra of free-base tetraphenylporphyrin and tetrakis(*p*-aminophenyl)porphyrin. The diprotonated forms have been modeled as their bis-formate complexes, i.e., as so-called porphyrin diacids. The dramatic redshift of the Q-band of the TAPP diacid has been explained in terms of an elevated “a_2u_” HOMO and lowered LUMOs, both reflecting infusion of aminophenyl character into the otherwise classic Gouterman-type frontier MOs. The exercise has also yielded valuable information on the performance of different exchange–correlation functionals. Thus, the hybrid B3LYP functional was found to yield a substantially better description of key spectral features, especially the diprotonation-induced redshifts, than the pure OLYP functional. Use of the range-separated CAMY-B3LYP functional, on the other hand, did not result in improvements relative to B3LYP.

## Introduction

**Figure 1:**
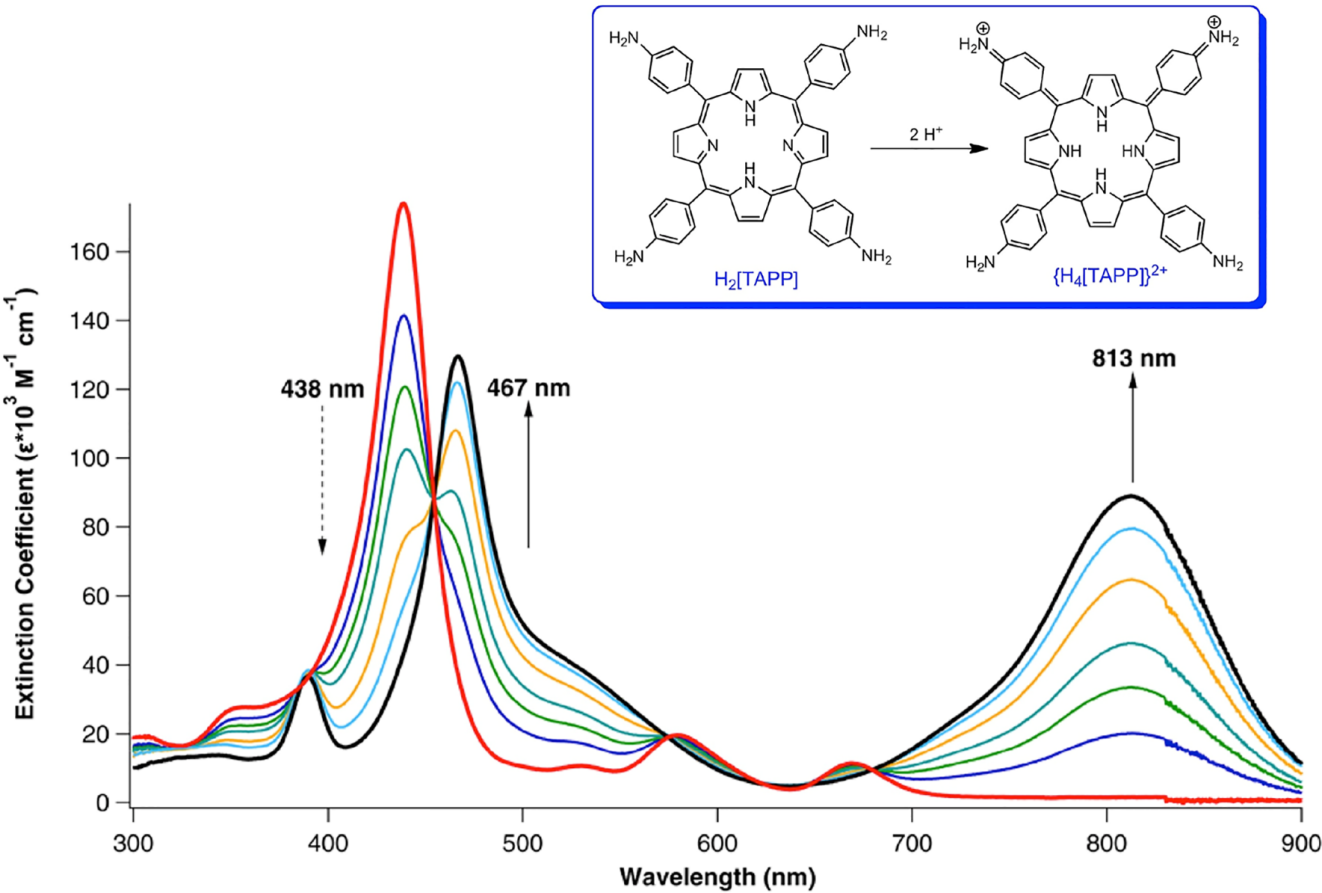
UV–vis–NIR spectral changes associated with titration of H_2_[TAPP] with methanesulfonic acid in DMSO. Adapted with permission from ref (2). Copyright 2014 American Chemical Society.

Porphyrins are notorious for leaving stains on glassware. Most tetraphenylporphyrins are dissolved by acid, which transforms their characteristic purple color to a brilliant green. The color change corresponding to diprotonation of tetraphenylporphyrin (H_2_[TPP]) is associated with modest redshifts of both the Soret and Q bands, from 417 and 646 nm to 443 and 659 nm, respectively.^1^ Much more dramatic spectral changes are observed for *meso*-tetrakis(*p*-aminophenyl)porphyrin (H_2_[TAPP]), for which protonation of the two unprotonated central nitrogens results in redshifts of the Soret and Q bands, originally at 438 and 669 nm in DMSO, to 467 and 813 nm, respectively (Figure 1).^2^ In addition to the redshift, the Q-band also dramatically gains in intensity. Martin Gouterman^3^ and co-workers, using semiempirical calculations, recognized these redshifts as a form of hyperporphyrin character,^4^ reflecting charge transfer from the *meso*-aryl groups.^5^ Modern quantum chemical methods, however, have not been applied to H_2_[TAPP] and its diprotonated form.^6−8^

As it happens, even unprotonated H_2_[TAPP] exhibits a number of peculiarities. Thus, unlike most *para*-substituted tetraphenylporphyrins, which exhibit optical spectra that are qualitatively indistinguishable from parent H_2_[TPP], H_2_[TAPP] exhibits significantly redshifted Soret and Q bands. Second, the oxidation potential of H_2_[TAPP] is substantially lower than that predicted on the basis of a Hammett correlation applicable to the great majority of *para*-substituted tetraphenylporphyrins.^2^ Taken together, these results suggest that the HOMO of H_2_[TAPP] is unexpectedly high in energy and that the amino substituent acts differently from other *para* substituents. Is H_2_[TAPP] itself to be viewed as an incipient hyperporphyrin?

**Chart 1:**
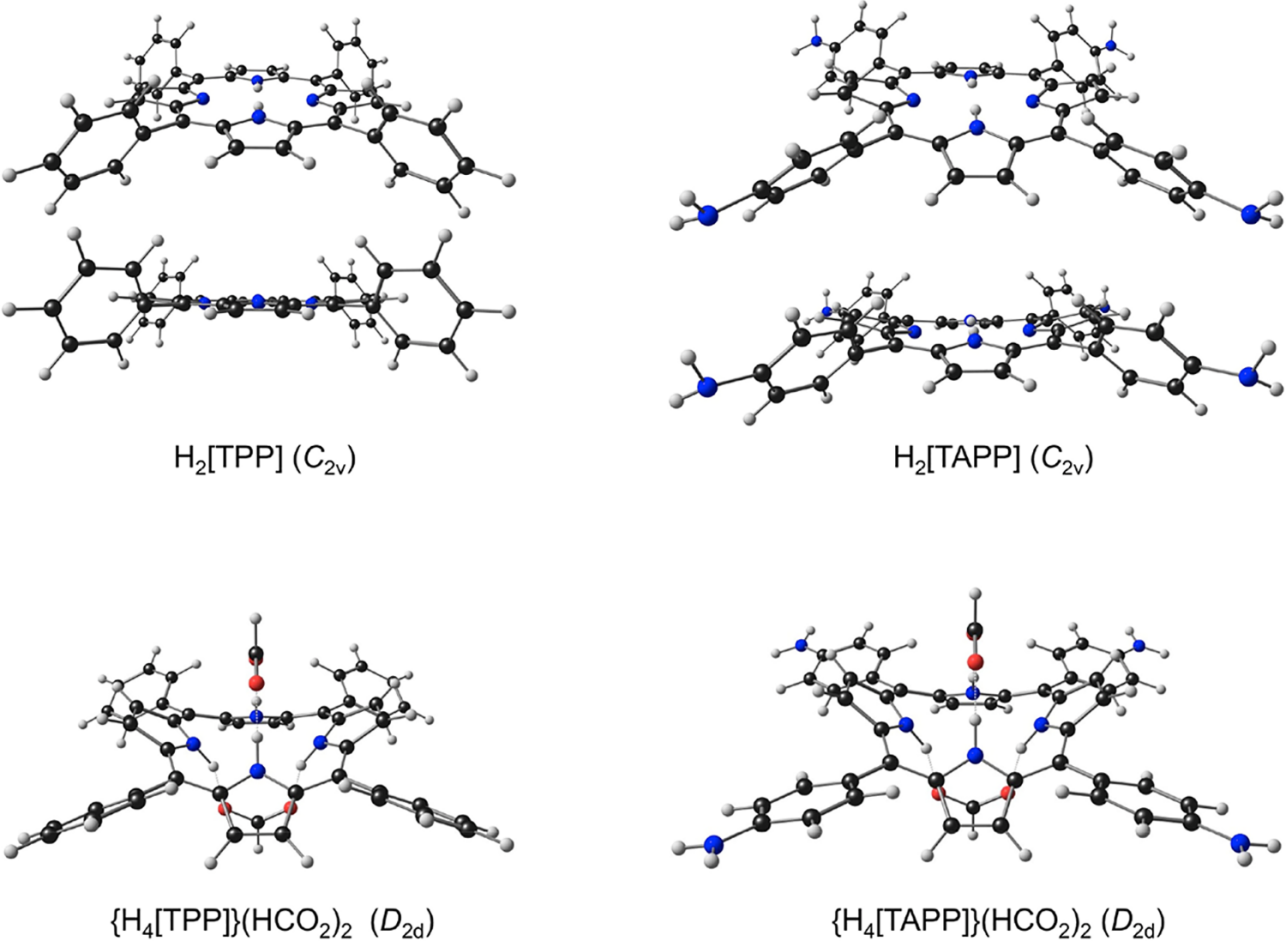
Molecules Studied in This Worka

The above phenomena, while of interest in and of themselves, are relevant to a number of practical applications. As dyes absorbing in the near-infrared (NIR), hyperporphyrins are clearly of interest in photomedicine, for example, as photosensitizers in photodynamic therapy.^9−13^ Protonated porphyrins have been used as sensors for gases such as ammonia, hydrogen sulfide, and sulfur dioxide,^14,15^ while H_2_[TAPP] has also been used as a building block for dye-sensitized solar cells.^16−18^ Porphyrin protonation has also been used for pH^19,20^ and anion sensing^21^ and even for modulating the optical properties of metal–organic frameworks (MOFs).^22^ Thus, motivated, we sought to shed light on the above spectral shifts via time-dependent (TD) density functional theory (DFT)^23^ calculations on H_2_[TPP], H_2_[TAPP] and their centrally diprotonated forms (Chart 1).^24−29^ Gratifyingly, the results have led to a host of long-awaited insights and spectral assignments, as recounted below.

a Ball-and-stick diagrams based on OLYP-D3/STO-TZ2P optimized geometries.

## Results

### a The Theoretical Model

The term “theoretical model” emphasizes the assumptions underlying our study and encompasses a number of aspects. A key aspect, obviously, concerns the exact chemical nature of the molecules studied, especially of the diprotonated forms of the porphyrins. Here we have modeled them as highly symmetric (*D*_2d_) bis-formate adducts. These adducts, also known as porphyrin diacids,^30,31^ are experimentally well-known and have been structurally characterized. These models also have the advantage of being charge-neutral, which should help stave off spurious transitions that may result in the presence of unbalanced charges (a relatively common issue in TDDFT calculations).

A second, related aspect concerns solvation, which also helps deter spurious transitions in TDDFT calculations. The results quoted below all refer to the COSMO^32^ solvation model and dichloromethane as solvent. Experimentally, both DMSO and dichloromethane have been used.^1,2,8^ We also examined the PCM^33^ model (with the Gaussian program) and found that it does not make much of a difference relative to COSMO.

**Figure 2:**
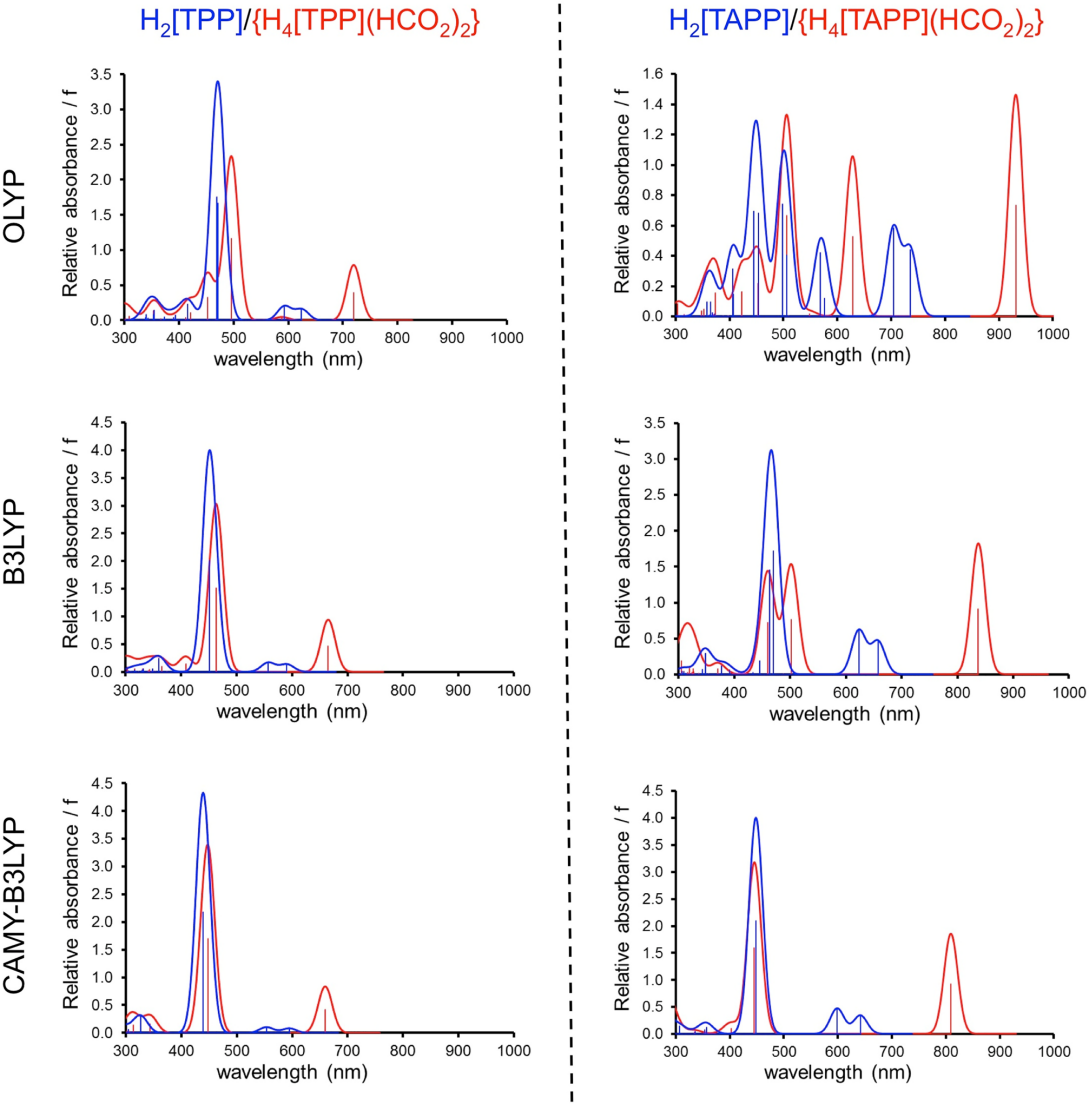
Calculated TDDFT UV–vis–NIR spectra in dichloromethane (COSMO) as a function of the exchange–correlation functional.

Finally, the choice of the exchange–correlation functional turned out to be important. We began our study using OLYP^34,35^-D3,^36^ OLYP being a generalized gradient approximation that has been extensively calibrated in our laboratory. The calculations indeed yielded valuable insights and assignments but also evinced a number of shortcomings that we wished to improve upon. Thus, OLYP predicted excessively large redshifts for the Q bands of the porphyrin diacids (Figure 2 andTable 2). The same calculations also predicted an intense transition (at 628 nm) between the main Soret and Q features of TAPP diacid, for which there does not appear to be an experimental counterpart (Figure 1).

**Table 1:** Comparison of TDDFT and Experimental^2,8^ Absorption Maxima (nm)

					experiment	
molecule	band	OLYP	B3LYP	CAMY-B3LYP	CH_2_Cl_2_	DMSO
H_2_[TPP]	Q	624.2	593.0	594.7	646	646
		592.6	558.5	553.6		
	Soret	471.5	417.1	439.3	416	417
		469.5	412.4	439.1		
{H_4_[TPP]}(HCO_2_)_2_	Q	719.7	657.3	660.0	657	659
	Soret	495.4	445.0	447.6	438	443
H_2_[TAPP]	Q	735.4	649.0	641.8	655	669
		704.0	612.5	598.8		
	Soret	576.6	447.9	448.3	427	438
		568.4	436.6	448.1		
{H_4_[TAPP]}(HCO_2_)_2_	Q	931.4	792.5	809.1	725	813
	Soret	506.1	490.0	445.1	460	466
			450.1			

**Table 2:** OLYP/STO-TZ2P TDDFT Results, Including Transition Energies (*E*) and Wavelengths (λ), Oscillator Strengths (*f*), MO Compositions, and Excited State Symmetries

					MO composition			
compound	peak	*E* (eV)	λ (nm)	*f*	from	to	weight (%)	state symmetry
H_2_[TPP]	Q	1.986	624.2	0.157	HOMO	LUMO	79.6	B_2_
					HOMO–1	LUMO+1	19.5	B_2_
		2.092	592.6	0.202	HOMO	LUMO+1	78.3	B_1_
					HOMO–1	LUMO	20.4	B_1_
	Soret	2.630	471.5	1.669	HOMO–1	LUMO+1	76.6	B_2_
					HOMO	LUMO	17.3	B_2_
		2.641	469.5	1.749	HOMO–1	LUMO	76.4	B_1_
					HOMO	LUMO+1	19.1	B_1_
{H_4_[TPP]}(HCO_2_)_2_	Q	1.723	719.7	0.394	HOMO	LUMO	88.9	E
					HOMO–1	LUMO	8.7	E
	Soret	2.503	495.4	1.165	HOMO–1	LUMO	75.6	E
					HOMO–7	LUMO	9.8	E
		2.742	452.2	0.323	HOMO–7	LUMO	89.2	E
					HOMO–1	LUMO	7.0	E
H_2_[TAPP]	Q	1.686	735.4	0.439	HOMO	LUMO	92.3	B_2_
		1.761	704.0	0.581	HOMO	LUMO+1	92.4	B_1_
	Soret	2.150	576.6	0.117	HOMO–1	LUMO	75.0	B_1_
					HOMO–4	LUMO	24.4	B_1_
		2.181	568.4	0.419	HOMO–1	LUMO+1	82.3	B_2_
					HOMO–4	LUMO+1	16.7	B_2_
		2.451	505.8	0.402	HOMO–5	LUMO	47.1	B_2_
					HOMO–4	LUMO+1	43.6	B_2_
					HOMO–1	LUMO+1	7.8	B_2_
		2.488	498.3	0.739	HOMO–4	LUMO	54.6	B_1_
					HOMO–5	LUMO+1	27.8	B_1_
					HOMO–1	LUMO	15.0	B_1_
		2.734	453.5	0.681	HOMO–5	LUMO	48.3	B_2_
					HOMO–4	LUMO+1	31.9	B_2_
					HOMO–7	LUMO	6.1	B_2_
					HOMO–1	LUMO+1	5.9	B_2_
		2.789	444.5	0.695	HOMO–5	LUMO+1	66.4	B_1_
					HOMO–4	LUMO	14.7	B_1_
					HOMO–1	LUMO	6.9	B_1_
					HOMO	LUMO+1	5.5	B_1_
		3.044	407.3	0.147	HOMO–7	LUMO+1	95.4	B_1_
		3.053	406.1	0.315	HOMO–7	LUMO	89.6	B_2_
{H_4_[TAPP]}(HCO_2_)_2_	Q	1.331	931.4	0.731	HOMO	LUMO	96.1	E
	Soret	1.972	628.6	0.526	HOMO–1	LUMO	98.1	E
		2.450	506.1	0.664	HOMO–4	LUMO	77.0	E
					HOMO–9	LUMO	18.7	E
		2.741	452.4	0.217	HOMO–9	LUMO	67.2	E
					HOMO–2,3	LUMO+2	12.7	E
					HOMO–4	LUMO	10.2	E
		2.931	423.0	0.164	HOMO–11	LUMO	89.0	E
					HOMO–2,3	LUMO+2	9.4	E

**Figure 3:**
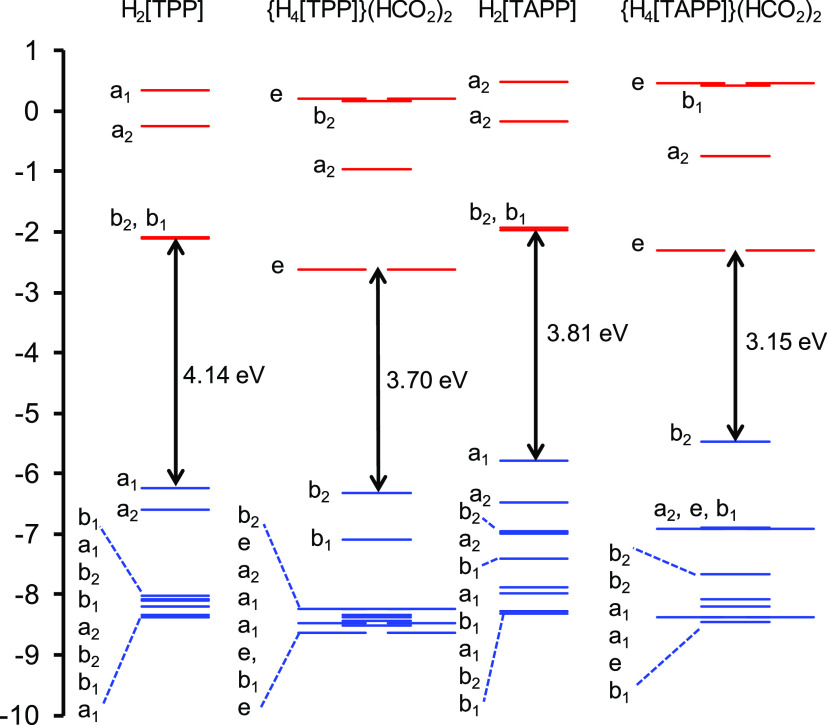
CAMY-B3LYP/STO-TZ2P Kohn–Sham MO energy (eV) level diagram for the four species studied, in dichloromethane modeled with COSMO.

The above problems were largely solved with B3LYP^37,38^ and its range-separated counterpart CAMY-B3LYP.^39−41^ Thus, both these functionals yielded Q-band redshifts that agreed well with experiment. Somewhat surprisingly, CAMY-B3LYP did not lead to improved results relative to B3LYP. We speculate that adjusting the amount of exchange in the B3LYP functional may well result in even better agreement between theory and experiment. Be that as it may, the present results, in our view, are entirely satisfactory and allow clear assignments for the protonation-induced spectral changes of H_2_[TPP] and H_2_[TAPP].

### b Molecular Orbital (MO) Energy Level Diagrams

**Figure 4:**
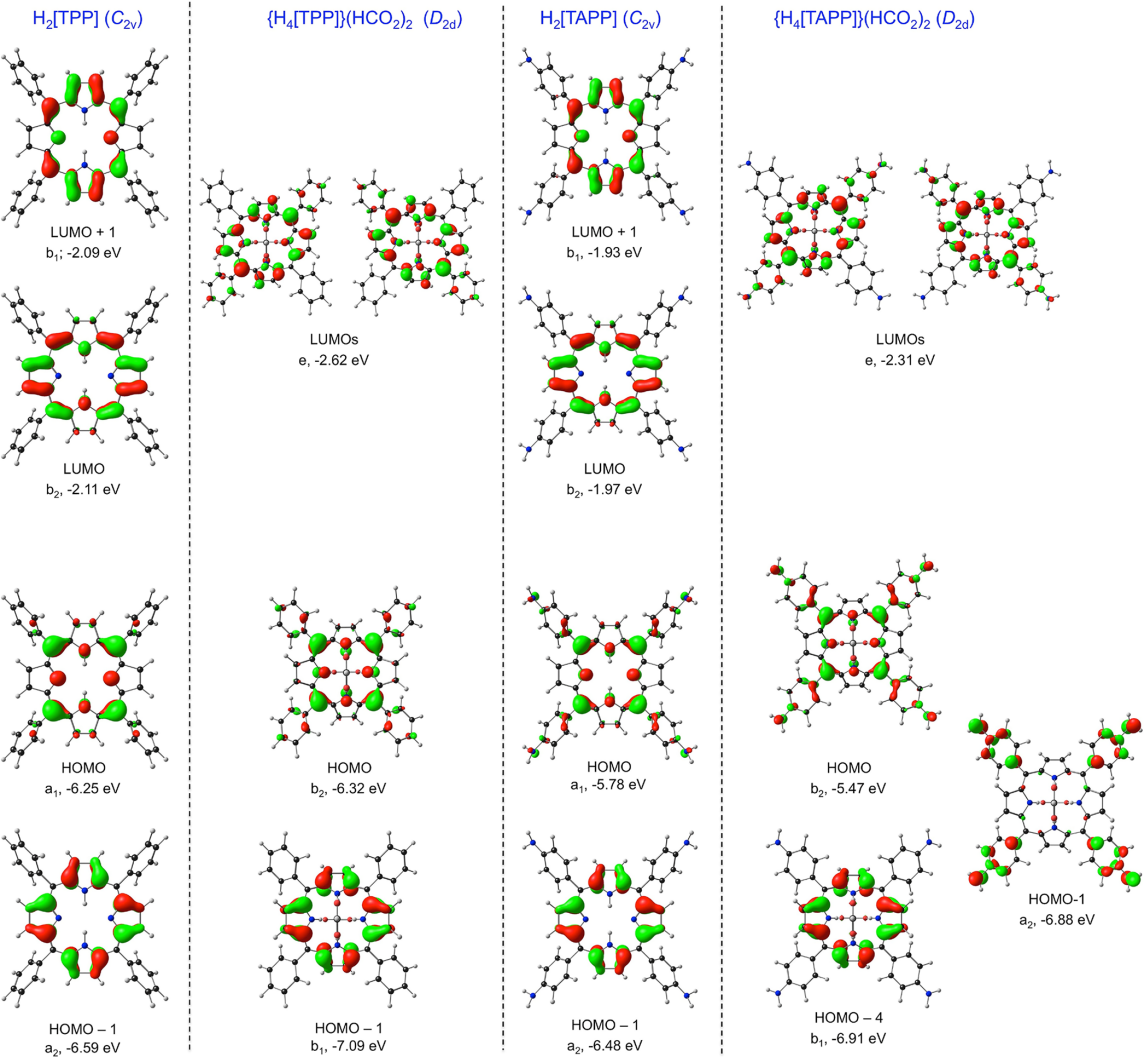
Key CAMY-B3LYP (COSMO) frontier MOs, along with their irreps and orbital energies, relevant to Figure 3.

A comparative Kohn–Sham molecular orbital (MO) energy level diagram (Figure 3) provides substantial insight into the observed spectral shifts and the hyperporphyrin effect and nicely sets the stage for a discussion of spectral assignments. The relevant MOs are depicted in Figure 4.

Of the four species examined, only H_2_[TPP] conforms *strictly* to the Gouterman four-orbital model;^42,43^ i.e., the two HOMOs are energetically close and the two LUMOs are essentially degenerate and these 4 MOs are energetically well-separated from all other MOs. That said, we shall see that the main optical transitions of all four species do conform to a largely Gouterman-type four-orbital composition. Below, although we will generally describe key MOs in terms of their actual irreducible representations (irreps), on occasion we will also use the well-known *D*_4*h*_ irreps, within quotation marks, to facilitate allusion to the four-orbital model.

For strongly saddled TPP diacid, the “a_2u_” HOMO (which transforms as b_2_ under *D*_2d_) drops marginally in energy relative to H_2_[TPP] (Figures 3 and 4). The drop appears to be associated with a slightly greater delocalization of the MO onto the phenyl groups relative to parent H_2_[TPP]. The LUMOs undergo a sharper drop, reflecting *substantial* delocalization onto the phenyl groups. These orbital energy shifts result in a significant contraction of the HOMO–LUMO gap, qualitatively explaining the Q-band redshift observed (11−13 nm, depending on the solvent; Table 1) upon protonation of H_2_[TPP]. The “a_1u_” HOMO (transforming as b_1_) also drops sharply, away from the “a_2u_” HOMO (Figure 3). These changes in orbital energy are best viewed as the combined effects of protonation, the resulting strong saddling, and enhanced porphyrin-phenyl conjugation as a result of the latter; it is unclear whether the individual effects of the three factors can be rigorously dissected into separate, additive contributions.

Compared with H_2_[TPP], H_2_[TAPP] exhibits a slight rise in the LUMO energy levels and a sharper rise in the energy of the “a_2u_” HOMO (Figure 3), understandably, given the large amplitudes of the latter MO at the *meso* positions (Figure 4). The result is again a contraction of the HOMO–LUMO gap, coincidentally to about the same value as for TPP diacid.

TAPP diacid exhibits LUMO energy levels slightly higher than those of TPP diacid, but the energy of the “a_2u_” HOMO (transforming, again, as b_2_) is considerably higher (Figures 3 and 4), reflecting the combined effects of protonation, strong saddling, and *para*-amino substitution. The HOMO–LUMO gap accordingly is dramatically contracted, qualitatively consistent with the extremely redshifted Q-band of TAPP diacid. Interestingly, although the “a_1u_” MO has approximately the same energy as that in TPP diacid, it corresponds to HOMO–4/HOMO–5 in TAPP diacid, depending on the functional. Between the “a_2u_” and “a_1u_” HOMOs, lie 2–3 aminophenyl-based MOs, disrupting the simple four-orbital model.

### c Spectral Assignments

Tables 2–4 present the detailed TDDFT data, including the MO-to-MO composition of key transitions for OLYP, B3LYP, and CAMY-B3LYP. Although the three functionals tell the same broad story, the reader may readily verify that the latter two functionals yield excitation energies (wavelengths) in significantly better agreement with experiment (Figure 1 and Table 1).

**Table 3:** B3LYP/STO-TZ2P TDDFT Results, Including Transition Energies (*E*) and Wavelengths (λ), Oscillator Strengths (*f*), MO Compositions, and Symmetries

					MO composition			
molecule	peak	*E* (eV)	λ (nm)	*f*	from	to	weight (%)	symmetry
H_2_[TPP]	Q	2.10	590.2	0.14	HOMO	LUMO	76	B2
					HOMO–1	LUMO+1	23	B2
		2.23	556.8	0.17	HOMO	LUMO+1	75	B1
					HOMO–1	LUMO	25	B1
	Soret	2.75	451.2	1.99	HOMO–1	LUMO+1	75	B2
					HOMO	LUMO	22	B2
		2.75	451.0	2.02	HOMO–1	LUMO	74	B1
					HOMO	LUMO+1	24	B1
{H_4_[TPP]}(HCO_2_)_2_	Q	1.86	665.3	0.47	HOMO	LUMO	89	E
					HOMO–1	LUMO	10	E
	Soret	2.68	463.1	1.51	HOMO–1	LUMO	87	E
					HOMO	LUMO	10	E
H_2_[TAPP]	Q	1.88	657.9	0.46	HOMO	LUMO	90	B2
					HOMO–1	LUMO+1	8	B2
		1.99	623.7	0.61	HOMO	LUMO+1	92	B1
					HOMO–1	LUMO	8	B1
	Soret	2.64	469.7	1.72	HOMO–1	LUMO+1	82	B2
					HOMO	LUMO	7	B2
		2.67	463.5	1.45	HOMO–1	LUMO	86	B1
					HOMO	LUMO+1	7	B1
{H_4_[TAPP]}(HCO_2_)_2_	Q	1.48	837.3	0.91	HOMO	LUMO	97	E
					HOMO–4	LUMO	2	E
	Ar → LUMO	2.47	501.6	0.76	HOMO–1	LUMO	94	E
					HOMO–4	LUMO	5	E
		2.54	487.4	0.009	HOMO–2,3	LUMO	100	B2
	Soret	2.70	459.6	0.72	HOMO–4	LUMO	85	E
					HOMO–5	LUMO	6	E
					HOMO–1	LUMO	5	E

**Table 4:** CAMY-B3LYP/STO-TZ2P TDDFT Results, Including Transition Energies (*E*) and Wavelengths (λ), Oscillator Strengths (*f*), MO Compositions, and Symmetries

					MO composition			
molecule	peak	*E* (eV)	λ (nm)	*f*	from	to	weight (%)	symmetry
H_2_[TPP]	Q	2.08	594.7	0.08	HOMO	LUMO	69	B_2_
					HOMO–1	LUMO+1	29	B_2_
		2.24	553.6	0.11	HOMO	LUMO+1	67	B_1_
					HOMO–1	LUMO	31	B_1_
	Soret	2.82	439.3	2.16	HOMO–1	LUMO+1	70	B_2_
					HOMO	LUMO	28	B_2_
		2.82	439.1	2.18	HOMO–1	LUMO	67	B_1_
					HOMO	LUMO+1	31	B_1_
{H_4_[TPP]}(HCO_2_)_2_	Q	1.88	660.0	0.42	HOMO	LUMO	85	E
					HOMO–1	LUMO	13	E
	Soret	2.77	447.6	1.70	HOMO–1	LUMO	84	E
					HOMO	LUMO	13	E
H_2_[TAPP]	Q	1.93	641.7	0.34	HOMO	LUMO	82	B_2_
					HOMO–1	LUMO+1	14	B_2_
		2.07	598.8	0.47	HOMO	LUMO+1	84	B_1_
					HOMO–1	LUMO	14	B_1_
	Soret	2.77	448.3	2.10	HOMO–1	LUMO+1	83	B_2_
					HOMO	LUMO	15	B_2_
		2.77	448.1	1.91	HOMO–1	LUMO	83	B_1_
					HOMO	LUMO+1	14	B_1_
{H_4_[TAPP]}(HCO_2_)_2_	Q	1.53	809.1	0.93	HOMO	LUMO	94	E
					HOMO–4	LUMO	3	E
	Soret	2.79	445.1	1.59	HOMO–4	LUMO	86	E
					HOMO–1	LUMO	7	E
		3.082	402.2	0.096	HOMO–1	LUMO	86.5	E
					HOMO–4	LUMO	7.2	E
		3.200	387.4	0.024	HOMO–2,3	LUMO	97.7	E

As expected, the Q and Soret transitions of H_2_[TPP] exhibit a classic Gouterman four-orbital composition (Table 1). Thus, the two near-degenerate Q features (Q_*x*_ and Q_*y*_) may be described as primarily HOMO → LUMO and HOMO → LUMO+1 transitions, while the two Soret features may be described as primarily HOMO–1 → LUMO and HOMO–1 → LUMO+1 transitions, respectively. In terms of composition, the Q and Soret bands of TPP diacid are also similar: the Q bands are thus essentially HOMO → LUMO (e), while the Soret bands are essentially HOMO–1 → LUMO (e), noting that the LUMOs are exactly degenerate in the *D*_2*d*_ diacids. The calculations generally do a good job of reproducing the protonation-induced redshifts of the Soret bands (experimentally about 22–26 nm, depending on the solvent) but somewhat overestimate the Q-band redshifts (with OLYP significantly worse than B3LYP and CAMY-B3LYP).

The two Q and Soret bands of H_2_[TAPP] are compositionally very similar to those of H_2_[TPP], i.e., being essentially HOMO → LUMO/LUMO+1 and HOMO–1 → LUMO/LUMO+1, respectively. The B3LYP and CAMY-B3LYP calculations do a good job of reproducing the modest redshifts of Q and Soret bands (experimentally about 11–21 nm, depending on the solvent) relative to H_2_[TPP]. Once again, OLYP greatly overestimates these observed redshifts.

The calculated, degenerate Q transitions of TAPP diacid may be described as essentially pure “a_2u_” → LUMO (e) transitions. The strongly redshifted position of the transition appears 2-fold in origin, an elevated “a_2u_” HOMO and lower-energy e LUMOs, relative to H_2_[TAPP]; both effects reflect infusion of aminophenyl character into the classic Gouterman-type frontier MO in question. The “a_1u_” HOMO of TAPP diacid, in contrast, is lower in energy, relative to H_2_[TAPP], which explains a modest protonation-induced redshift for the Soret band. Interestingly, while B3LYP does a good job of reproducing the observed Soret redshift, CAMY-B3LYP predicts a small blueshift instead.

A major difference between B3LYP and CAMY-B3LYP concerns the Soret region of TAPP diacid. Thus, while B3LYP predicts two Soret-like features at 459.6 and 501.6 nm (Figure 2 and Table 3), CAMY-B3LYP predicts a unique, dominant Soret maximum at 445.1 nm (Figure 2 and Table 4). As indicated in Table 3, the 501.1 nm peak with B3LYP is a degenerate pair of aminophenyl → LUMO (e) transitions. Such transitions also occur with CAMY-B3LYP, but with much weaker intensities and on the higher-energy side of the major Soret peak (Table 4).

## Conclusion

A first TDDFT study of tetraphenylporphyrin and tetrakis(*p*-aminophenyl)porphyrin diacids has afforded substantial insight into the origin of their hyperporphyrin spectra.^45^ In short, multiple effects account for hyperporphyrin spectra.

Two different effects are primarily responsible for the Q-band redshifts. For diacid formation, the major contributor to the Q-band redshifts is a lowering of the LUMOs as a result of infusion of *meso*-aryl character. Elevation of the “a_2u_” HOMO also plays a role, albeit a smaller one. In contrast, the redshifted Q-band of H_2_[TAPP] relative to H_2_[TPP] reflects destabilization of the “a_2u_” HOMO via interaction with aminophenyl-based occupied MOs, while the LUMOs remain energetically relatively unperturbed.

Beyond the Q bands (i.e., for the Soret bands as well as certain pre-Soret and post-Soret bands), the transitions of the diacid forms are compositionally more complex. In these, *meso*-aryl → LUMO character mixes in with classic Gouterman “a_1u_” → LUMO transitions. Indeed, some of these transitions may be described as primarily *meso*-aryl or aminophenyl-based; the intensities of these transitions appear to vary significantly with the exchange–correlation functional.

An important finding, from a methodological point of view, is that the hybrid functionals B3LYP and CAMY-B3LYP perform much better than the pure functional OLYP. Use of the range-separated CAMY-B3LYP functional, however, does not appear to confer any significant advantage relative to classic B3LYP. Additional functionals, as well as solvent effects, are being examined in our laboratory. Overall, the above study has led to straightforward insights into an important class of hyperporphyrin spectra, which, we believe, should significantly aid in the design of a variety of porphyrin-based functional materials such as phototherapeutics, sensors, and solar dyes.

## Computational Methods

All calculations were carried out with the ADF^44^ 2018 program with all-electron ZORA-STO-TZ2P basis sets, fine meshes for numerical integration of matrix elements, and adequately tight convergence criteria for both SCF and geometry optimization cycles. Molecular geometries were optimized with OLYP^34,35^-D3^36^ with appropriate symmetry constraints (as indicated in Chart 1e); these optimized geometries were then used for TDDFT calculations with the OLYP-D3, B3LYP,^37,38^ and CAMY-B3LYP^39^ functionals. The COSMO^32^ solvation model (with dichloromethane as solvent) was used throughout.

## Supporting Information Available

Optimized Cartesian coordinates (PDF)
