# Supplementary material for: Understanding Hyperporphyrin Spectra: TDDFT Calculations on Diprotonated Tetrakis(p-aminophenyl)porphyrin
Source: J Phys Chem A. 2021 Oct 29;125(46):9953–61. doi: 10.1021/acs.jpca.1c06621 (PMC8630795; doi:10.1021/acs.jpca.1c06621)
Supplement: Supplementary file 1 — jp1c06621_si_001.pdf [file jp1c06621_si_001.pdf]

Supporting information

Understanding Hyperporphyrin Spectra: TDDFT Calculations on Diprotonated Tetrakis(*p*-aminophenyl)porphyrin

Jeanet Conradie,*[,a,b] Carl C. Wamser*[,c] and Abhik Ghosh*[,a]

[a] Department of Chemistry, UiT – The Arctic University of Norway, N-9037 Tromsø, Norway;

[b]Department of Chemistry, University of the Free State, P.O. Box 339, Bloemfontein 9300, Republic of South Africa.

[c]Department of Chemistry, Portland State University, Portland, Oregon 97207-0751

Email: conradj@ufs.ac.za (JC); wamserc@pdx.edu (CCW); abhik.ghosh@uit.no (AG)

OLYP-D3/ZORA-STO-TZ2P optimized Cartesian coordinates (Å)

Table of Contents

1. [H_2TPP]; C_{2v} S2
2. [H_4TPP]$(HCOO)_2$; D_{2d} S3
3. [H_2TAPP]; C_{2v} S5
4. [H_4TAPP]$(HCOO)_2$; D_{2d} S7

Optimized OLYP/ZORA-STO-TZ2P Cartesian coordinates (Å)

1. [H_2TPP]; C_{2v}

C	0.685865000	4.257423000	0.073700000
C	0.685865000	-4.257423000	0.073700000
C	1.132133000	2.898645000	-0.015098000
C	1.132133000	-2.898645000	-0.015098000
C	2.460854000	2.444530000	-0.020735000
C	2.460854000	-2.444530000	-0.020735000
C	2.863717000	1.091347000	-0.032162000
C	2.863717000	-1.091347000	-0.032162000
C	3.519124000	3.492905000	-0.015118000
C	3.519124000	-3.492905000	-0.015118000
C	3.636391000	4.395558000	-1.081735000
C	3.636391000	-4.395558000	-1.081735000
C	4.256624000	0.679282000	-0.148972000
C	4.256624000	-0.679282000	-0.148972000
C	4.413108000	3.597765000	1.060085000
C	4.413108000	-3.597765000	1.060085000
C	4.626220000	5.378642000	-1.076083000
C	4.626220000	-5.378642000	-1.076083000
C	5.400459000	4.583230000	1.069690000
C	5.400459000	-4.583230000	1.069690000
C	5.510922000	5.476933000	0.000985000
C	5.510922000	-5.476933000	0.000985000
C	-0.685865000	4.257423000	0.073700000
C	-0.685865000	-4.257423000	0.073700000
C	-1.132133000	2.898645000	-0.015098000
C	-1.132133000	-2.898645000	-0.015098000
C	-2.460854000	2.444530000	-0.020735000
C	-2.460854000	-2.444530000	-0.020735000
C	-2.863717000	1.091347000	-0.032162000
C	-2.863717000	-1.091347000	-0.032162000
C	-3.519124000	3.492905000	-0.015118000
C	-3.519124000	-3.492905000	-0.015118000
C	-3.636391000	4.395558000	-1.081735000
C	-3.636391000	-4.395558000	-1.081735000
C	-4.256624000	0.679282000	-0.148972000
C	-4.256624000	-0.679282000	-0.148972000
C	-4.413108000	3.597765000	1.060085000
C	-4.413108000	-3.597765000	1.060085000
C	-4.626220000	5.378642000	-1.076083000
C	-4.626220000	-5.378642000	-1.076083000
C	-5.400459000	4.583230000	1.069690000
C	-5.400459000	-4.583230000	1.069690000
C	-5.510922000	5.476933000	0.000985000
C	-5.510922000	-5.476933000	0.000985000
H	0.000000000	1.105799000	-0.081385000
H	0.000000000	-1.105799000	-0.081385000

H	1.343584000	5.109952000	0.142004000
H	1.343584000	-5.109952000	0.142004000
H	2.945859000	4.319471000	-1.916914000
H	2.945859000	-4.319471000	-1.916914000
H	4.326516000	2.901194000	1.889023000
H	4.326516000	-2.901194000	1.889023000
H	4.707449000	6.067450000	-1.914050000
H	4.707449000	-6.067450000	-1.914050000
H	5.102701000	1.344726000	-0.238042000
H	5.102701000	-1.344726000	-0.238042000
H	6.082685000	4.655253000	1.913874000
H	6.082685000	-4.655253000	1.913874000
H	6.280875000	6.245067000	0.007914000
H	6.280875000	-6.245067000	0.007914000
H	-1.343584000	5.109952000	0.142004000
H	-1.343584000	-5.109952000	0.142004000
H	-2.945859000	4.319471000	-1.916914000
H	-2.945859000	-4.319471000	-1.916914000
H	-4.326516000	2.901194000	1.889023000
H	-4.326516000	-2.901194000	1.889023000
H	-4.707449000	6.067450000	-1.914050000
H	-4.707449000	-6.067450000	-1.914050000
H	-5.102701000	1.344726000	-0.238042000
H	-5.102701000	-1.344726000	-0.238042000
H	-6.082685000	4.655253000	1.913874000
H	-6.082685000	-4.655253000	1.913874000
H	-6.280875000	6.245067000	0.007914000
H	-6.280875000	-6.245067000	0.007914000
N	0.000000000	2.117730000	-0.065751000
N	0.000000000	-2.117730000	-0.065751000
N	2.034836000	0.000000000	0.026880000
N	-2.034836000	0.000000000	0.026880000

2. $[H_4TPP](HCOO)_2$; D_{2d}

C	0.000000000	0.000000000	3.057700000
C	0.000000000	0.000000000	-3.057700000
C	0.000000000	3.453500000	0.000000000
C	0.000000000	4.928000000	0.000000000
C	0.000000000	7.739600000	0.000000000
C	0.000000000	-3.453500000	0.000000000
C	0.000000000	-4.928000000	0.000000000
C	0.000000000	-7.739600000	0.000000000
C	1.061700000	7.038300000	-0.578700000
C	1.061700000	-7.038300000	0.578700000
C	1.064400000	5.645500000	-0.576500000
C	1.064400000	-5.645500000	0.576500000
C	1.194600000	2.780100000	0.329200000
C	1.194600000	-2.780100000	-0.329200000
C	2.330100000	3.301300000	1.027900000
C	2.330100000	-3.301300000	-1.027900000

C	2.780100000	1.194600000	0.329200000
C	2.780100000	-1.194600000	-0.329200000
C	3.301300000	2.330100000	1.027900000
C	3.301300000	-2.330100000	-1.027900000
C	3.453500000	0.000000000	0.000000000
C	4.928000000	0.000000000	0.000000000
C	5.645500000	1.064400000	-0.576500000
C	5.645500000	-1.064400000	0.576500000
C	7.038300000	1.061700000	-0.578700000
C	7.038300000	-1.061700000	0.578700000
C	7.739600000	0.000000000	0.000000000
C	-1.061700000	7.038300000	0.578700000
C	-1.061700000	-7.038300000	-0.578700000
C	-1.064400000	5.645500000	0.576500000
C	-1.064400000	-5.645500000	-0.576500000
C	-1.194600000	2.780100000	-0.329200000
C	-1.194600000	-2.780100000	0.329200000
C	-2.330100000	3.301300000	-1.027900000
C	-2.330100000	-3.301300000	1.027900000
C	-2.780100000	1.194600000	-0.329200000
C	-2.780100000	-1.194600000	0.329200000
C	-3.301300000	2.330100000	-1.027900000
C	-3.301300000	-2.330100000	1.027900000
C	-3.453500000	0.000000000	0.000000000
C	-4.928000000	0.000000000	0.000000000
C	-5.645500000	1.064400000	0.576500000
C	-5.645500000	-1.064400000	-0.576500000
C	-7.038300000	1.061700000	0.578700000
C	-7.038300000	-1.061700000	-0.578700000
C	-7.739600000	0.000000000	0.000000000
H	0.000000000	0.000000000	4.176500000
H	0.000000000	0.000000000	-4.176500000
H	0.000000000	8.827100000	0.000000000
H	0.000000000	-8.827100000	0.000000000
H	1.036700000	1.036700000	-0.864900000
H	1.036700000	-1.036700000	0.864900000
H	1.880200000	5.100200000	-1.040300000
H	1.880200000	-5.100200000	1.040300000
H	1.885700000	7.578100000	-1.039200000
H	1.885700000	-7.578100000	1.039200000
H	2.364800000	4.273500000	1.496300000
H	2.364800000	-4.273500000	-1.496300000
H	4.273500000	2.364800000	1.496300000
H	4.273500000	-2.364800000	-1.496300000
H	5.100200000	1.880200000	-1.040300000
H	5.100200000	-1.880200000	1.040300000
H	7.578100000	1.885700000	-1.039200000
H	7.578100000	-1.885700000	1.039200000
H	8.827100000	0.000000000	0.000000000
H	-1.036700000	1.036700000	0.864900000
H	-1.036700000	-1.036700000	-0.864900000

H	-1.880200000	5.100200000	1.040300000
H	-1.880200000	-5.100200000	-1.040300000
H	-1.885700000	7.578100000	1.039200000
H	-1.885700000	-7.578100000	-1.039200000
H	-2.364800000	4.273500000	-1.496300000
H	-2.364800000	-4.273500000	1.496300000
H	-4.273500000	2.364800000	-1.496300000
H	-4.273500000	-2.364800000	1.496300000
H	-5.100200000	1.880200000	1.040300000
H	-5.100200000	-1.880200000	-1.040300000
H	-7.578100000	1.885700000	1.039200000
H	-7.578100000	-1.885700000	-1.039200000
H	-8.827100000	0.000000000	0.000000000
N	1.485700000	1.485700000	-0.029200000
N	1.485700000	-1.485700000	0.029200000
N	-1.485700000	1.485700000	0.029200000
N	-1.485700000	-1.485700000	-0.029200000
O	0.801000000	0.801000000	-2.506900000
O	0.801000000	-0.801000000	2.506900000
O	-0.801000000	0.801000000	2.506900000
O	-0.801000000	-0.801000000	-2.506900000

3. [H_2TAPP]; C_{2v}

C	0.686817000	4.215792000	0.414302000
C	0.686817000	-4.215792000	0.414302000
C	1.133166000	2.890844000	0.110498000
C	1.133166000	-2.890844000	0.110498000
C	2.461256000	2.446284000	-0.025697000
C	2.461256000	-2.446284000	-0.025697000
C	2.845260000	1.092160000	-0.172372000
C	2.845260000	-1.092160000	-0.172372000
C	3.488242000	4.578614000	-0.882192000
C	3.488242000	-4.578614000	-0.882192000
C	3.520239000	3.481141000	-0.005863000
C	3.520239000	-3.481141000	-0.005863000
C	4.194968000	0.680395000	-0.524352000
C	4.194968000	-0.680395000	-0.524352000
C	4.481896000	5.549184000	-0.867554000
C	4.481896000	-5.549184000	-0.867554000
C	4.595800000	3.399805000	0.894406000
C	4.595800000	-3.399805000	0.894406000
C	5.550249000	5.466311000	0.046076000
C	5.550249000	-5.466311000	0.046076000
C	5.589032000	4.370136000	0.928917000
C	5.589032000	-4.370136000	0.928917000
C	-0.686817000	4.215792000	0.414302000
C	-0.686817000	-4.215792000	0.414302000
C	-1.133166000	2.890844000	0.110498000
C	-1.133166000	-2.890844000	0.110498000

C	-2.461256000	2.446284000	-0.025697000
C	-2.461256000	-2.446284000	-0.025697000
C	-2.845260000	1.092160000	-0.172372000
C	-2.845260000	-1.092160000	-0.172372000
C	-3.488242000	4.578614000	-0.882192000
C	-3.488242000	-4.578614000	-0.882192000
C	-3.520239000	3.481141000	-0.005863000
C	-3.520239000	-3.481141000	-0.005863000
C	-4.194968000	0.680395000	-0.524352000
C	-4.194968000	-0.680395000	-0.524352000
C	-4.481896000	5.549184000	-0.867554000
C	-4.481896000	-5.549184000	-0.867554000
C	-4.595800000	3.399805000	0.894406000
C	-4.595800000	-3.399805000	0.894406000
C	-5.550249000	5.466311000	0.046076000
C	-5.550249000	-5.466311000	0.046076000
C	-5.589032000	4.370136000	0.928917000
C	-5.589032000	-4.370136000	0.928917000
H	0.000000000	1.121943000	-0.175600000
H	0.000000000	-1.121943000	-0.175600000
H	1.344086000	5.046969000	0.617618000
H	1.344086000	-5.046969000	0.617618000
H	2.675291000	4.658185000	-1.598146000
H	2.675291000	-4.658185000	-1.598146000
H	4.439920000	6.380890000	-1.567806000
H	4.439920000	-6.380890000	-1.567806000
H	4.643631000	2.561332000	1.583125000
H	4.643631000	-2.561332000	1.583125000
H	5.012166000	1.347133000	-0.758467000
H	5.012166000	-1.347133000	-0.758467000
H	6.402654000	4.290424000	1.646967000
H	6.402654000	-4.290424000	1.646967000
H	6.622007000	7.022246000	-0.724907000
H	6.622007000	-7.022246000	-0.724907000
H	7.394952000	6.198972000	0.520188000
H	7.394952000	-6.198972000	0.520188000
H	-1.344086000	5.046969000	0.617618000
H	-1.344086000	-5.046969000	0.617618000
H	-2.675291000	4.658185000	-1.598146000
H	-2.675291000	-4.658185000	-1.598146000
H	-4.439920000	6.380890000	-1.567806000
H	-4.439920000	-6.380890000	-1.567806000
H	-4.643631000	2.561332000	1.583125000
H	-4.643631000	-2.561332000	1.583125000
H	-5.012166000	1.347133000	-0.758467000
H	-5.012166000	-1.347133000	-0.758467000
H	-6.402654000	4.290424000	1.646967000
H	-6.402654000	-4.290424000	1.646967000
H	-6.622007000	7.022246000	-0.724907000
H	-6.622007000	-7.022246000	-0.724907000
H	-7.394952000	6.198972000	0.520188000

H	-7.394952000	-6.198972000	0.520188000
N	0.000000000	2.124660000	-0.042842000
N	0.000000000	-2.124660000	-0.042842000
N	2.033618000	0.000000000	0.003915000
N	6.507911000	6.470468000	0.115657000
N	6.507911000	-6.470468000	0.115657000
N	-2.033618000	0.000000000	0.003915000
N	-6.507911000	6.470468000	0.115657000
N	-6.507911000	-6.470468000	0.115657000

4. [H_4TAPP](HCOO)$_2$; D_{2d}

C	0.000000000	0.000000000	3.116054000
C	0.000000000	0.000000000	-3.116054000
C	0.000000000	3.457341000	0.000000000
C	0.000000000	4.916108000	0.000000000
C	0.000000000	7.764083000	0.000000000
C	0.000000000	-3.457341000	0.000000000
C	0.000000000	-4.916108000	0.000000000
C	0.000000000	-7.764083000	0.000000000
C	1.153971000	5.654291000	-0.348734000
C	1.153971000	-5.654291000	0.348734000
C	1.160703000	7.037187000	-0.352683000
C	1.160703000	-7.037187000	0.352683000
C	1.175683000	2.765788000	0.383662000
C	1.175683000	-2.765788000	-0.383662000
C	2.251334000	3.225838000	1.200383000
C	2.251334000	-3.225838000	-1.200383000
C	2.765788000	1.175683000	0.383662000
C	2.765788000	-1.175683000	-0.383662000
C	3.225838000	2.251334000	1.200383000
C	3.225838000	-2.251334000	-1.200383000
C	3.457341000	0.000000000	0.000000000
C	4.916108000	0.000000000	0.000000000
C	5.654291000	1.153971000	-0.348734000
C	5.654291000	-1.153971000	0.348734000
C	7.037187000	1.160703000	-0.352683000
C	7.037187000	-1.160703000	0.352683000
C	7.764083000	0.000000000	0.000000000
C	-1.153971000	5.654291000	0.348734000
C	-1.153971000	-5.654291000	-0.348734000
C	-1.160703000	7.037187000	0.352683000
C	-1.160703000	-7.037187000	-0.352683000
C	-1.175683000	2.765788000	-0.383662000
C	-1.175683000	-2.765788000	0.383662000
C	-2.251334000	3.225838000	-1.200383000
C	-2.251334000	-3.225838000	1.200383000
C	-2.765788000	1.175683000	-0.383662000
C	-2.765788000	-1.175683000	0.383662000

C	-3.225838000	2.251334000	-1.200383000
C	-3.225838000	-2.251334000	1.200383000
C	-3.457341000	0.000000000	0.000000000
C	-4.916108000	0.000000000	0.000000000
C	-5.654291000	1.153971000	0.348734000
C	-5.654291000	-1.153971000	-0.348734000
C	-7.037187000	1.160703000	0.352683000
C	-7.037187000	-1.160703000	-0.352683000
C	-7.764083000	0.000000000	0.000000000
H	0.000000000	0.000000000	4.237341000
H	0.000000000	0.000000000	-4.237341000
H	0.829671000	9.644584000	-0.240257000
H	0.829671000	-9.644584000	0.240257000
H	1.069226000	1.069226000	-0.892753000
H	1.069226000	-1.069226000	0.892753000
H	2.049551000	5.120022000	-0.648861000
H	2.049551000	-5.120022000	0.648861000
H	2.058600000	7.577480000	-0.644625000
H	2.058600000	-7.577480000	0.644625000
H	2.246736000	4.157887000	1.745914000
H	2.246736000	-4.157887000	-1.745914000
H	4.157887000	2.246736000	1.745914000
H	4.157887000	-2.246736000	-1.745914000
H	5.120022000	2.049551000	-0.648861000
H	5.120022000	-2.049551000	0.648861000
H	7.577480000	2.058600000	-0.644625000
H	7.577480000	-2.058600000	0.644625000
H	9.644584000	0.829671000	-0.240257000
H	9.644584000	-0.829671000	0.240257000
H	-0.829671000	9.644584000	0.240257000
H	-0.829671000	-9.644584000	-0.240257000
H	-1.069226000	1.069226000	0.892753000
H	-1.069226000	-1.069226000	-0.892753000
H	-2.049551000	5.120022000	0.648861000
H	-2.049551000	-5.120022000	-0.648861000
H	-2.058600000	7.577480000	0.644625000
H	-2.058600000	-7.577480000	-0.644625000
H	-2.246736000	4.157887000	-1.745914000
H	-2.246736000	-4.157887000	1.745914000
H	-4.157887000	2.246736000	-1.745914000
H	-4.157887000	-2.246736000	1.745914000
H	-5.120022000	2.049551000	0.648861000
H	-5.120022000	-2.049551000	-0.648861000
H	-7.577480000	2.058600000	0.644625000
H	-7.577480000	-2.058600000	-0.644625000
H	-9.644584000	0.829671000	0.240257000
H	-9.644584000	-0.829671000	-0.240257000
N	0.000000000	9.126971000	0.000000000
N	0.000000000	-9.126971000	0.000000000
N	1.502292000	1.502292000	-0.047654000
N	1.502292000	-1.502292000	0.047654000

N	9.126971000	0.000000000	0.000000000
N	-1.502292000	1.502292000	0.047654000
N	-1.502292000	-1.502292000	-0.047654000
N	-9.126971000	0.000000000	0.000000000
O	0.802165000	0.802165000	-2.569171000
O	0.802165000	-0.802165000	2.569171000
O	-0.802165000	0.802165000	2.569171000
O	-0.802165000	-0.802165000	-2.569171000
